# Unravelling the Mysteries of the Sonic Hedgehog Pathway in Cancer Stem Cells: Activity, Crosstalk and Regulation

**DOI:** 10.3390/cimb46060323

**Published:** 2024-05-29

**Authors:** Carlo Berrino, Aadilah Omar

**Affiliations:** Division of Oncology, Department of Internal Medicine, Faculty of Health Sciences, University of the Witwatersrand, Johannesburg 2193, South Africa

**Keywords:** Sonic Hedgehog, cancer stem cells, signalling pathways

## Abstract

The Sonic Hedgehog (Shh) signalling pathway plays a critical role in normal development and tissue homeostasis, guiding cell differentiation, proliferation, and survival. Aberrant activation of this pathway, however, has been implicated in the pathogenesis of various cancers, largely due to its role in regulating cancer stem cells (CSCs). CSCs are a subpopulation of cancer cells with the ability to self-renew, differentiate, and initiate tumour growth, contributing significantly to tumorigenesis, recurrence, and resistance to therapy. This review focuses on the intricate activity of the Shh pathway within the context of CSCs, detailing the molecular mechanisms through which Shh signalling influences CSC properties, including self-renewal, differentiation, and survival. It further explores the regulatory crosstalk between the Shh pathway and other signalling pathways in CSCs, highlighting the complexity of this regulatory network. Here, we delve into the upstream regulators and downstream effectors that modulate Shh pathway activity in CSCs. This review aims to cast a specific focus on the role of the Shh pathway in CSCs, provide a detailed exploration of molecular mechanisms and regulatory crosstalk, and discuss current and developing inhibitors. By summarising key findings and insights gained, we wish to emphasise the importance of further elucidating the interplay between the Shh pathway and CSCs to develop more effective cancer therapies.

## 1. Introduction

### 1.1. The Sonic Hedgehog (Shh) Pathway and Its Significance in Normal Development and Tissue Homeostasis

The Shh pathway has garnered significant attention primarily because of its crucial involvement in developmental processes and embryogenesis. The Shh ligand is a highly active regulator that belongs to a family of proteins that induce Hh signalling. In mammals, there are three Hh proteins: Sonic Hedgehog (Shh), Indian Hedgehog (Ihh), and Desert Hedgehog (Dhh). Shh and Ihh share crucial roles across various tissues, with some overlapping functions [1]. Specifically, Shh is prominently involved in specifying cell types within the nervous system and in the patterning of limbs. In contrast, Ihh plays a significant role in the development of the skeletal system, especially in the process of endochondral ossification. Dhh expression is confined to the reproductive system, including the granulosa cells in ovaries and Sertoli cells in testes. Among these, the role of Shh in mouse embryogenesis has been extensively studied, particularly its function in neural progenitor patterning. This process involves the differentiation of six distinct cell types, identifiable by molecular markers, such as progenitors of interneurons and motor neurons, which occur through a gradient of Shh signalling. The Shh signalling pathway plays a crucial role in the formation and development of numerous tissues and organs, including the eyes, limbs, hair, skin, teeth, central nervous system (CNS), cochlea, and lungs, and is essential for establishing tissue polarity [2,3].While the Shh pathway is frequently suppressed in adults, it plays a role in maintaining somatic stem cells and epithelial cells as well as being involved in tissue repair [4]. Shh signalling maintains neural stem cells to allow for the constant development of new neurons [5]. Shh signalling is also required for the maintenance of chondrocytes and satellite cells, the somatic stem cells of bone and muscle tissue, respectively, to allow for cell regeneration after injury [5].

### 1.2. Canonical and Non-Canonical Pathways

The operational dynamics of the Shh pathway can manifest through either a canonical or a non-canonical mechanism. Canonical activation of the Hh-GLI signalling pathway is initiated when Hh ligands attach to the twelve-pass transmembrane receptor Patched-1 (PTCH1) [6]. This binding releases the inhibition on the seven-pass transmembrane G protein-coupled receptor Smoothened (SMO). As a result, SMO becomes active and triggers a series of intricate intracellular events that culminate in the activation of the three GLI transcription factors, which serve as the ultimate effectors of the Hh-GLI pathway [7]. Traditionally, it has been believed that vertebrate canonical and non-canonical Hh signalling pathways operate independently and within separate cellular compartments [8]. The bulk of research has centred on the canonical pathway of Hh signalling, highlighting its reliance on primary cilia, which are microtubule-based structures acting as signalling centres. However, a growing body of research points to the significant roles played by non-canonical Hh signalling. Furthermore, accumulating evidence suggests a complex relationship between canonical and non-canonical Hh signalling pathways, indicating that they may not only share physical domains but also interact and influence each other functionally in various instances [9].

**Figure 1:**
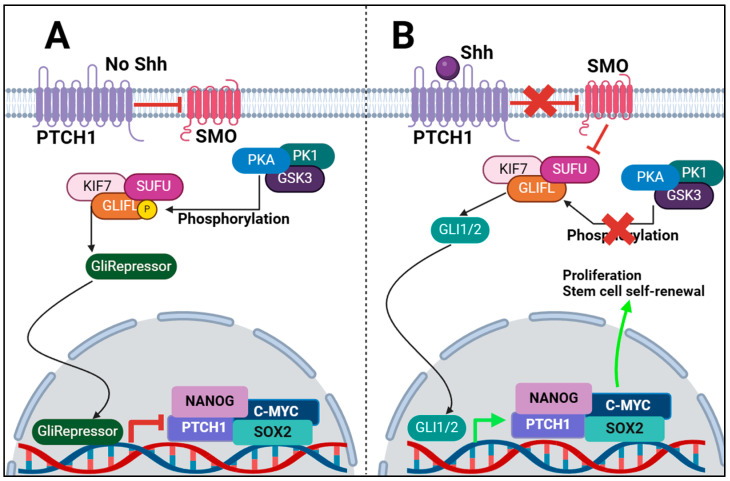
The canonical Shh signalling pathway. (**A**) When no Shh ligand is present, the signalling pathway is inactive. PTCH1 binds to SMO, inhibiting it and preventing it from binding to SUFU. SUFU binds GLIFL and KIF7. A protein complex consisting of PKA, PK1 and GSK3 binds to and phosphorylates GLIFL. SUFU then cleaves GLIFL to GliRepressor consisting of GLI2/3 proteins. GliRepressor moves into the nucleus and inhibits transcription of Shh/Gli1 signalling genes. (**B**) When Shh ligand is present, it binds to PTCH1, inhibiting its ability to inactivate SMO. SMO then binds to SUFU which is bound to KIF7 and GLIFL. SMO inhibits SUFU to prevent phosphorylation of GLIFL, allowing KIF7 to detach from the complex and migrate to the cilia. GLIFL cleaved into GLI1/2. The GLI1/2 protein then migrates into the nucleus and initiates transcription and translation of *PTCH1*, *NANOG*, *SOX2*, *c-MYC* and other Shh related genes. These proteins then maintain the stem cell properties of the cell, enabling replicative immortality. The red X indicates the inhibition of SMO and the Phosphorylation of GLIFL is being inhibited.

Without its ligand, PTCH1 is positioned at the primary cilium’s base, a cellular extension emerging from vertebrate cells and acting as a dedicated hub for Hh signalling processes [10]. The primary cilium plays a crucial role in processing various cellular signals and/or changes in the extracellular environment that are essential for animal development. This is achieved via several signalling pathways such as Wingless (Wnt), Platelet-derived growth factor (PDGF), Shh, and Notch [11]. The initiation of Hh signalling via the canonical pathway is triggered by the attachment of Hh ligands to the PTCH1 receptor, as shown in Figure 1. When no ligands are present, PTCH1 binds to SMO, preventing its accumulation in the organelle and inhibiting its function. In this state, GLI2 and GLI3 transcription factors are phosphorylated by protein kinase A (PKA), casein kinase 1 (CK1), and glycogen synthase kinase 3β (GSK3β). Following phosphorylation, these factors are recognised by the β-TrCP protein and are retained in the cytoplasm through interactions with Kinesin family member 7 (KIF7) and Suppressor of Fused (SUFU), which inhibits their transport to the nucleus by binding to the base of the primary cilia [12,13,14]. While in the cytoplasm, the phosphorylated GLI2 and GLI3 are cleaved, generating truncated versions that inhibit the transcription of Hh-responsive genes [15]. GLI1, in contrast, does not undergo this repressive cleavage but is mainly governed by transcriptional regulation and further regulated through the ubiquitin–proteasome system (UPS). This system targets two specific degradation signals within GLI1, blocking inappropriate signalling [16] (Figure 1A).

The Hh-GLI pathway becomes active when the Hh ligand binds and relocates PTCH1 away from the primary cilium, leading to its internalisation into the cytoplasm and degradation by lysosomes [6]. This event enables SMO to be phosphorylated and activated via its association with CK1α and GPCR kinase 2 (GRK2), and translocation to the cilium [17]. Once activated, SMO transmits the Hh signal that halts the processing of GLI2 and GLI3 and facilitates their separation from SUFU and the KIF7 motor protein. In the presence of Shh ligand, KIF7 migrates to the tip of the primary cilium, away from SUFU and preventing phosphorylation of GLI1/2 by PKA [14]. Consequently, this allows for the active, full-length GLI proteins to enter the nucleus and activate the transcription of Hh target genes, namely, *NANOG*, *SOX2*, *PTCH1*, and *C-MYC*, shown in Figure 1B. These genes play roles in cellular proliferation, survival, renewal, and invasion. Notably, GLI1 serves as a dependable marker of Hh pathway activation in its own right and depends on active GLI2 and GLI3 for its transcriptional activation and expression [18], thus completing a regulatory feedback loop [19,20].

Non-canonical signalling is categorised into two main types: Type I is any form of signalling that induces activity of GLI1/2 without the involvement of SMO, most commonly caused by signalling crosstalk. Type II proceeds through SMO, following the normal Shh signalling pathway, but leads to crosstalk with other signalling pathways such as MAPK, leading to cell processes that are not directly controlled by Shh signalling [21]. Thus, while numerous non-canonical Hh signalling pathways may incorporate the traditional elements PTCH1 and SMO, they diverge by not solely triggering GLI activation [22]. Instead, they initiate a variety of alternative downstream responses.

### 1.3. Abnormalities in Shh Signalling

#### 1.3.1. Birth Defects

The Hh signalling pathway plays a critical role in various processes during embryonic development, such as the formation and organisation of the neural tube [23]. A deficiency in the Shh ligand can lead to holoprosencephaly (HPE), a midline defect, while elevated Shh signalling is linked to conditions like exencephaly and spina bifida [24]. Adding complexity to this relationship is the fact that mutations in proteins necessary for the functioning of cilia frequently result in weakened Shh signalling and interfere with the proper closure of the neural tube. Shh pathway mutations have been linked to human HPE, and both natural and synthetic inhibitors of Shh signalling have been shown to cause HPE and orofacial clefts in mice. Human malformation-associated mutations have been reported in the *Shh* gene itself, the genes encoding the Shh secretory protein (*DISP1*), the Shh receptor (*PTCH1*) and associated membrane proteins (*CDON*, *BOC*, and *GAS1*), and *GLI2*, the dominant pathway transcriptional activator [25]. Furthermore, studies have shown abnormal Hh signalling within the palatal mesenchyme interferes with the oral–nasal organisation of the neural crest cell-derived ectomesenchyme in the palatal shelves. This results in impaired formation of the palatine bone and a completely penetrant occurrence of cleft palate [26]. During the development of the skeleton, Shh and Ihh signals play crucial roles by providing positional cues by initiating or sustaining cellular differentiation processes that govern the development of cartilage and bone. Any dysfunction in the Hh signalling pathway can lead to significant skeletal abnormalities [27].

#### 1.3.2. Cancer

Elevated Shh expression has been linked to enhanced cell division, epithelial–mesenchymal transition (EMT), and cell mobility, all of which contribute to tumour growth and treatment resistance. Evidence shows that dysregulation of the Shh pathway accelerates the development of various cancers, such as colorectal [28], gastric [29], pancreatic [30], and prostate cancers [31]. Continuous activation of the Hh-GLI signalling pathway has been observed in both solid tumours and blood-related malignancies. This ongoing activation is linked to the initiation, advancement, and recurrence of tumours following chemotherapy, primarily through its role in controlling the surviving CSCs [32]. Research involving model organisms and human subjects has significantly enhanced our understanding of Hh signalling pathways, uncovering its involvement in numerous human cancers.

Disruption in Hh-GLI signalling can occur through several mechanisms: (a) Ligand independent signalling, where activation does not require an Hh ligand to bind to PTCH1 to initiate Hh signalling. This can be caused by loss-of-function mutations in PTCH1 or SUFU, gain-of-function mutations in SMO, or amplifications of GLI genes, as seen in conditions like basal cell carcinoma (BCC), Shh-subtype medulloblastoma, and rhabdomyosarcoma (RMS). (b) Ligand-dependent activation in an autocrine manner, where cancer cells have a mutation that leads to overexpression of Hh ligands, leading to the cells both producing and responding to Hh ligands independently, observed in glioma, melanoma, and cancers of the lung, breast, stomach, and prostate. (c) Paracrine ligand-dependent activation, in which cancer cells oversecrete Hh ligands that activate Hh signalling in adjacent stromal cells. This stromal activation, in turn, promotes the tumour’s growth and survival, and this effect is mutual, seen in pancreatic and colorectal cancer (CRC) [33,34].

### 1.4. Introduction to CSCs and Their Pivotal Role in Tumorigenesis, Recurrence, and Resistance to Therapy

#### 1.4.1. Overview

Research has identified that many tumours are composed of two distinct types of cells: differentiated and undifferentiated [35]. These variations may account for differences in tumour biology and behaviour. Studies have highlighted that the undifferentiated subgroup of cancer cells shares similarities with normal adult stem cells, including the innate ability for self-renewal and the potential to evolve into progenitor cells [36]. The cancer stem cell hypothesis posits that within a tumour, there exists a minor subset of cells with primitive characteristics similar to those of somatic stem cells. These cells, known as CSCs, possess the unique ability to differentiate into all the various cell types found within the tumour [37,38]. These particular cells are characterised by their capability to initiate and drive tumour growth and progression, thereby earning the classification as CSCs. There is a growing body of evidence supporting the role of CSCs in cancer development, revealing that a small population of these stem cells can maintain the tumour’s existence, promote its growth, and lead to the formation of diverse cancer cell populations [39]. In addition to these cells possessing the properties of differentiation and self-renewal, these subpopulations of cells are known to influence therapeutic response and clinical outcomes [40].

#### 1.4.2. Metastatic Stem Cells

Recent findings also suggest that CSCs exhibit enhanced mobility, enabling them to penetrate nearby tissues and facilitate metastasis to distant organs [41]. Plasticity in the process of EMT facilitates the formation of CSCs at various stages of metastasis, including the phase of metastatic colonisation. The latter stages of metastasis, specifically initiation and colonisation, present significant challenges for disseminated tumour cells (DTCs) in evolving into detectable metastases [42]. Consequently, only a select few DTCs, known as “metastasis-initiating cells” (MICs) or “metastatic stem cells” (MetSCs), possess the necessary adaptability and cancer stem cell-like characteristics to start tumour growth in new, distant tissues [43]. These cells mirror the CSCs found in the original tumour, equipped with the capacity to initiate tumour growth across various tissues. To do so, they employ a range of survival tactics, including metabolic adaptation, stromal co-option, immune evasion, and resistance to therapy [43]. Additionally, the processes of EMT and its counterpart, mesenchymal–epithelial transition (MET), serve as a link between metastasis and CSCs, further highlighting the critical role CSCs in the spread of cancer [44,45].

Despite the importance of CSCs in metastasis, research in this area remains nascent, hindered by methodological difficulties in tracking the progression of CSCs, or newly formed CSCs, during the metastatic process. Progress in understanding the regulatory networks that control the epithelial–mesenchymal plasticity (EMP) and/or CSC characteristics of cells has led to the creation of computational models. These models help unravel the complex behaviour of these states, generate new hypotheses, and may pinpoint potential interventions to limit cellular plasticity. The introduction of cutting-edge single-cell analytical technologies, such as mass cytometry (CyTOF), intravital microscopy, lineage tracing, and other advanced techniques, has opened up new avenues for gaining detailed insights into how spatial and temporal dynamics, as well as cell heterogeneity, play roles in metastasis formation [46,47]. By employing sophisticated computational strategies to analyse these rich, high-dimensional data, researchers can better understand the variability and dynamics of EMP and/or CSC features [48]. This approach facilitates the formulation of novel hypotheses for experimental validation. The cycle of using mathematical models to interpret experimental and clinical findings, and vice versa, promises to shed light on the complex and nonlinear behaviour characteristics of MetSCs [49,50].

Stem cell-related genes such as *SOX2*, *NANOG*, *KLF4*, *OCT4*, *SNAI2*, *SNAI1*, *SOX9*, and others are significant promoters of stemness and contribute to increased metastasis in various cancer types [49,51]. These genes have also been observed in clinical metastases. Recent research in breast cancer has revealed that clusters of circulating tumour cells (CTCs) with metastatic potential exhibit hypomethylation of stemness-related transcription factors, including *SOX2*, *NANOG*, *OCT4*, and *SIN3A*. Their methylation status inversely correlates with metastasis, as a loss of their expression diminishes stemness-associated functions [52]. Furthermore, the methylation patterns of these genes in CTC clusters have been linked to poorer prognoses. Additional instances include the expression of *SOX2/SOX9* in DTCs, which leads to the initiation of metastasis following a latency period in breast cancer, as well as the promotion of metastasis by *SLUG/SOX9* and the stemness regulator miR-199a in both normal and cancer cells, favouring the initiation of tumours and metastases [53,54,55].

#### 1.4.3. CSC Markers

The cell surface glycoprotein known as Cluster of Differentiation 44 (CD44) plays a critical role in various cellular functions, including cell cohesion, differentiation, movement, and proliferation. CD44 was identified as the initial marker of CSCs across various cancer types, and a high prevalence of CD44-positive cells is closely associated with tumour recurrence and increased malignancy [56,57]. CD44, especially its variant isoform CD44v, acts as a marker for CSCs not merely due to its specific presence on the cell surface but crucially because of its significant role in controlling CSC characteristics. This regulation encompasses everything from the assimilation and relay of signals from the tumour environment and within the cell to the nucleus for self-renewal, to shielding the cell against damage or apoptosis caused by reactive oxygen species (ROS) or other harmful stimuli [58]. The increased resistance to ROS provides CSCs with a survival benefit, thereby fuelling tumour growth, metastasis, and resistance to chemotherapy [59]. CD44 is recognised as a characteristic surface marker for CSCs, often used alone or alongside other markers like CD24, CD133, CD34, and c-Met, to isolate or concentrate CSCs in different cancer forms [58].

#### 1.4.4. CSC Markers and Shh

The activation or inhibition of certain signalling pathways influences the expression of CD133 CSC marker, a target gene within these networks [60]. Malfunctions within these pathways lead to various abnormalities in CD133-positive cells, including altered self-renewal capacities, enhanced proliferation, disrupted differentiation, increased resistance to apoptosis and chemotherapy, elevated invasiveness, greater potential for metastasis, and a higher likelihood of cancer recurrence [61].

The clinical significance and the association between CSC markers and the Hh pathway in various cancer types is yet to be fully understood. A study examined the connection between Shh expression and established CSC markers (CD44, CD133, and *SOX2*) within oral squamous cell carcinoma, aiming to ascertain their influence on the clinical outcomes of these patients. SHH expression showed a significant correlation with CD133, and both Shh and *SOX2* expressions were linked to poorer survival rates. This study demonstrates that the overexpression of Shh is closely tied to CSC markers, playing a role in tumour advancement and leading to unfavourable patient outcomes [62]. Research has also indicated that positive immunohistochemical staining for CD44, Shh, and Gli1 proteins was associated with larger tumour sizes, more severe gross and histological types, and higher TNM stages. This correlation also indicated shorter overall survival and disease-free survival following tumour resection [63]. It was concluded that combined, CD44 and Shh signalling pathways are critical indicators of tumour severity, patient survival, and the risk of recurrence in gastric cancer.

In liver cancer, the Shh signalling pathway is found to be overactive in CD133-positive CSC’s, playing a critical role in preserving CSC characteristics. Consequently, targeting the Shh signalling pathway may offer a promising approach to combat liver cancer [64]. In the context of glioma, CD133-positive CSCs exhibit an increased resistance to chemotherapy. Specifically, the combined activity of the Shh and Notch signalling pathways enhances the resistance of CD133-positive CSCs to treatment with temozolomide (TMZ). Following TMZ treatment, there is a significant increase in the activity of both the Notch and Shh pathways in CD133-positive glioma cells, leading to the upregulation of Notch1 and GLI1. Thus, blocking the Shh and Notch pathways might improve the efficacy of TMZ therapy against CD133-positive glioma stem cells [65].

## 2. Shh Pathway in CSCs: Key Molecular Components of the Shh Pathway and Their Interactions within the Context of CSCs

As with most signalling pathways involved in cell proliferation and tissue repair, disruption of the Hh signalling pathway is known to lead to cancer [66]. PTCH1 plays the role of a tumour suppressor, as it inhibits the release of transcription factors. If its expression is disrupted or if it loses its function, then it will lead to constitutive signalling of SMO, an oncoprotein, and unimpeded release of GLI1/2 into the nucleus. This form of disruptive signalling is known as ligand-independent signalling, as the Hh signalling is constitutively active without the need of Shh ligand to bind to PTCH1 [67]. Another way ligand-independent signalling can occur is through a loss of function mutation of SUFU, as it also acts as a tumour suppressor. SUFU is essential to produce GLI repressor, which prevents GLI1/2 from being produced and moving into the cytoplasm. A loss of function mutation in SUFU leads to constitutive expression of GLI1/2 and Hh signalling target genes [68]. If SMO is overexpressed or receives an activating mutation, ligand-independent signalling can also occur, as SMO is able to function and initiate GLI1/2 release without signalling from Shh.

Other forms of Hh-disruptive signalling involve mutations in Shh secretion, leading to overexpression of Shh ligand and constitutively active Hh signalling through both autocrine and paracrine signalling mechanisms. These disruptions are known as ligand-dependent, as Shh ligand is still required for Hh signalling to occur [69,70]. Dispatched transporter family member 1 (DISP1) is a key protein involved in the secretion of Shh ligand, particularly long-range signalling. Deletion of the DISP1 gene led to accumulation of Shh ligand in source cells, leading to reduced Shh signalling [71]. Disruptions in DISP1 function could lead to excessive Shh secretion and ligand-dependent signalling in target cells. Another protein essential for secretion and transport of Shh ligand is Hedgehog acetyltransferase (HHAT). It plays a role in the post-translational palmitoylation of Shh ligand, and knockout studies showed decreased Shh ligand secretion and subsequently Shh signalling [72,73]. Similarly, disruptions in HHAT function could lead to increased Shh expression and signalling.

Stem cells are known for their ability to perpetuate themselves through self-renewal and maintaining pluripotency. Certain signalling pathways can stimulate cell differentiation into specific tissue types [74]. CSCs have the same abilities as normal stem cells; however, they have mutations in their genes that induce and propagate cancer. This is a problem, as it allows for CSCs to constantly induce tumour formation after the initial population of tumour cells has been eradicated [75].

The exact mechanism behind Shh maintaining the CSC self-renewal capabilities is unknown; however, studies performed on thyroid cancer [76,77,78,79], embryonal RMS [80], and breast cancer [81] revealed that increased Shh and SMO signalling lead to increased expression of *SOX2*, *NANOG*, *OCT3/4*, and *KLF4* transcription factors. These are the core transcription factors essential for maintaining the stem cell niche and self-renewal by inhibiting signalling pathways that induce cell differentiation. *OCT3/4* primarily maintains the pluripotent state and *NANOG* expression in both stem cells and CSCs, while *SOX2* and *NANOG* work to maintain expression of *OCT3/4*, which simultaneously maintains *NANOG* expression in a positive feedback loop [33,82]. This allows for the CSCs to maintain their genetic profiles, which contain mutations that lead to constitutively active proliferative signalling.

Signalling pathways play essential roles in regulating stem cell functions and are tightly controlled in normal stem cells. It is not surprising that these stemness signalling pathways operate aberrantly in CSCs. Numerous signalling pathways known for regulating self-renewal in normal stem cells have been implicated in promoting stemness and the progression of malignancies [83,84]. The JAK/STAT, Wnt/β-catenin, Notch, TGF-β, PI3K/AKT, Hh, and NF-kB pathways are crucial stemness signalling pathways involved in promoting and sustaining the stem-like attributes of CSCs [85].

In addition to cell proliferation, Shh is linked to the maintenance of stem cell characteristics. Key alterations in this pathway in cancer development include mutations in Shh components or the disruption of Shh ligand release. Notably, mutations in human tumours have been identified that impact various elements of the Shh pathway. These include loss of function mutations in PTCH1 or SUFU and gain of function mutations in GLI1, SMO, and GLI2, leading to ligand-independent activation of the pathway [33]. Tumour cells can alter their surrounding environment by emitting effector ligands that activate the Shh pathway in stromal cells, thereby regulating CSC formation and persistence across different tumours [86]. This regulation is achieved through the activation of essential stem cell-related genes like *BIM1*, *OCT4*, *SOX2*, and *NANOG*. Consequently, Shh signalling is pivotal in the development of drug resistance in many cancers and targeting this pathway may offer a promising approach to curb tumour growth, as well as to prevent relapse and resistance to treatment.

Studies have illustrated that targeting cancer cells alone is often insufficient due to the resilience of CSCs [87]. Investigating the interactions between CSCs and their microenvironment, and designing therapies aimed at these relationships, could lead to a reduction in CSC populations and better clinical outcomes for patients with metastatic cancers. The significance of Shh signalling in supporting the microenvironment and sustaining colorectal CSCs was explored in research by Fan et al. [88]. A particular focus has been on tumour-associated macrophages (TAMs) within the tumour microenvironment, known to foster CSC self-renewal and maintenance. Recent findings suggest that TAMs can contribute to CRC progression and resistance to therapy, both in lab models and in human patients [89]. The interaction between Stat3 and Shh signalling in CSCs has been implicated in these processes. According to Fan et al., TAMs can enhance the CSC phenotype by activating the Shh-GLI pathway in cancer cells, which, in turn, increases resistance to chemotherapy [88].

## 3. Crosstalk between the Shh Pathway and Other Signalling Pathways in the Regulation of CSC Properties

**Figure 2:**
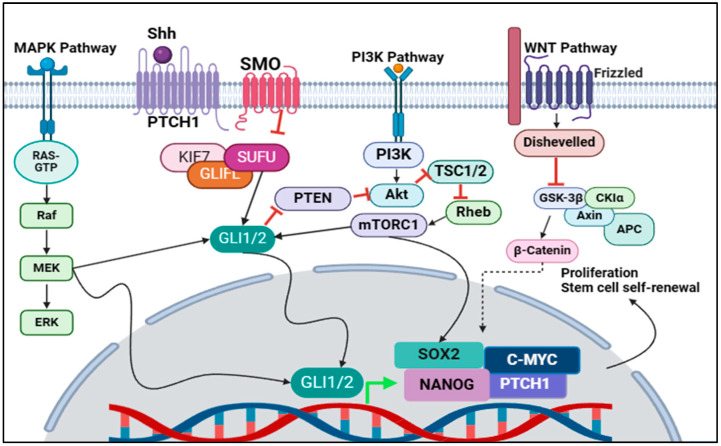
Crosstalk with the Shh signalling pathway. Cross talk is a phenomenon that occurs when multiple signalling pathways can affect the same protein and lead to similar effects. Alongside the normal Shh signalling pathway, expression of GLI1/2 can be regulated by MAPK/MEK signalling as well as PI3K/AKT/mTOR signalling. MEK is known to induce GLI1/2 signalling, while PI3K maintains a positive feedback loop through GLI1/2. PI3K signalling induces GLI1/2 expression which in turn leads to inhibition of PTEN, an inhibitor of AKT protein, which is essential for PI3K signalling. PI3K signalling is also known to increase *SOX2* expression directly. The exact mechanism in which WNT signalling has crosstalk with Shh signalling is unknown (Indicated by the dotted arrow); however, Shh-related genes (*SOX2/NANOG/PTCH1* and *C-MYC*) have been found to be upregulated in the presence of WNT signalling. This crosstalk with multiple pathways poses problems, as inhibition of the Shh signalling pathway can be bypassed by alternative signalling pathways. This is seen particularly when targeting SMO, an upstream regulator of Shh signalling, as GLI1/2 appears to be the common link between canonical and non-canonical (crosstalk) Shh signalling.

Shh signalling is a primary regulator of stemness and CSC properties; however, there is crosstalk between the Shh signalling pathway and various other pathways that could potentially cause resistance to Shh inhibition strategies, as shown in Figure 2.

### 3.1. MAPK/Shh Crosstalk

Various extracellular stimuli, including growth factors, osmotic stress, UV irradiation, ROS, cytokines, and integrins, induce the MAPK signalling pathway [76,90]. This pathway comprises three parallel branches: the classical MAPK, JNK, and p38 kinase pathways [91,92]. Activation of these pathways involves a cascade of multiple serine/threonine kinases in the sequence of MAP3K → MAP2K → MAPK. Specifically, the classical MAPK pathway is triggered by the binding of growth factors or cytokines to their receptor tyrosine kinases, which then activate Ras via two adaptor proteins, Grb2 and SOS [91]. Activation of Ras initiates the MAPK cascade, progressing as RAF (B-Raf, C-Raf, Raf-1) → MEK → ERK1/2 [91].

Numerous studies have indicated that activation of the MAPK pathway can amplify GLI1 transcriptional activity [93], leading to crosstalk between MAPK and Shh signalling. A study investigated NIH3T3 cells that were transfected with constitutively active MEK mutants, inducing aberrant signalling [94]. The study demonstrated that the expression of GLI target genes, including *GLI1* and *PTCH1*, was significantly increased in the presence of the MEK mutant. The same study found that MEK increases GLI1 transcription activity, and that GLI1 requires a functional NH_2_-terminal domain to respond to the MEK stimulus, marking a potential inhibitory site [94]. It is unknown whether MEK induces phosphorylation of the GLI1 functional NH_2_-terminal domain. Subsequent research suggested that ERK2 may play a role in phosphorylating a consensus site in the N-terminus of GLI1; however, it is not yet confirmed [95]. Inhibition of the MAPK pathway led to a significant decrease in Shh signalling activity and GLI1 target protein expression, potentially inhibiting CSC self-renewal activity. Similarly, Ji et al. investigated the inhibition of the MAPK pathway using the MEK inhibitor U0126 [96]. They found that it reduced GLI1 stability and dampened GLI1-mediated transcriptional activity in a pancreatic cancer cell line harbouring KRAS mutations. Additionally, inhibition of the MAPK pathway resulted in the suppression of GLI1 transcriptional activity in an HT-29 colon cancer cell line [97,98]. Simultaneous inhibition of both the Shh and MAPK pathways synergistically inhibited the proliferation of TE-1 gastric cancer cells [99]. GLI1 appears to have the most significant role in crosstalk between MAPK and Shh signalling for the maintenance of CSC stemness and tumorigenesis, and these studies showed that inhibition of just Shh or MAPK signalling did not entirely abolish GLI1 activity due to this crosstalk. Knowing the bridge between the signalling pathways potentially allows for the development of therapeutic strategies that target GLI1 as a bottleneck in CSC signalling.

### 3.2. PI3K/Shh Crosstalk

The PI3K/AKT/mTOR signalling pathway stands out as one of the central and most widely recognised pathways in various cell types. PI3Ks are intracellular enzymes responsible for phosphorylating hydroxyl groups on the inositol ring to generate PIP3 [100]. Among the various types of PI3K enzymes, the class I enzymes can activate protein kinase B (AKT). Activation of tyrosine kinases occurs with the binding of growth factors to their receptors, subsequently activating PI3K [101]. This activation cascade then triggers AKT and mTOR, which facilitate cell division and regulate various cellular processes. Many studies have investigated the PI3K/AKT/mTOR signalling pathway in maintaining CSCs and driving tumour progression. For example, inhibition of mTOR has been shown to diminish the activity of aldehyde dehydrogenase 1 (ALDH1), which is a validated biomarker for colorectal CSCs [102]. Recent studies have confirmed the supportive role of ALDH1 in stem cell differentiation and maintenance. Enhanced ALDH1 activity has been linked to increased stemness properties in CRC cells. Additionally, ALDH1 promotes CSC generation, and its upregulation and heightened activity are associated with a poor prognosis in CRC patients [103].

A study investigating the link between PI3K signalling and cancer stem cell activity found that the PI3K/mTOR pathway is a direct regulator of *SOX2* expression in colorectal CSCs. SOX2 is a protein target expressed during Shh/GLI1 signalling that induces and maintains stemness in CSCs [104]. The study found that PI3K/mTOR signalling initiated CSC activity through SOX2 expression when exposed to radiation, inducing radiation resistance in the colorectal CSCs [105]. They examined the effects of inhibiting PI3K signalling when irradiating the cells and saw that it was essential for SOX2 expression and CSC activity. This highlights the PI3K/mTOR pathway as a potential for bypassing radiation resistance in colorectal CSCs.

Another study found that PI3K upregulates GLI1/2 expression in renal cell carcinomas, allowing for the CSCs to bypass Hh/SMO signalling inhibition, marking a potential form of treatment resistance [106]. Interestingly, Zhou et al. also investigated the link between PI3K and CSC activity and found that GLI1 upregulated PI3K signalling as opposed to PI3K upregulating GLI1 [107]. They proposed that GLI1 induces CSC resistance to treatment through activation of the PI3K pathway. It could be hypothesised that PI3K upregulates GLI1 and GLI1 increases PI3K signalling in a positive feedback loop, to maintain stemness and resistance to treatment.

### 3.3. Wnt/Shh Crosstalk

The link between Wnt signalling and Shh signalling is poorly understood. There is no known direct interaction between the two pathways; however, some studies investigating Wnt found that it leads to the increased expression of CSC markers, *ATOH1*, *CCND1*, *CD44*, *FGF20*, *JAG1*, *LGR5*, *MYC*, and *SNAI1* [108]. Another study investigating Wnt signalling-induced breast tumours found that Shh signalling was also upregulated, showing increased expression of GLI1, PTCH1, and PTCH2 [109]. Studies investigating LGR4 (leucine-rich repeat-containing G-protein-coupled receptor) found that it upregulates Wnt signalling in prostate CSCs, while also increasing Shh and Notch signalling [110]. In LGR4-deficient cells, Shh and Notch signalling were downregulated, as well as the Shh target genes GLI1 and c-MYC. Another paper investigating LGR4 found that it increases the expression of SOX2, an Shh stem cell protein, through the Wnt/β-catenin/LEF signalling pathway. This led to increased mammary regeneration potential in mammary stem cells, highlighting it as another potential site of crosstalk between the pathways [111]. While LGR4 shows a potential crosstalk role between the pathways, it is still unclear whether there is direct crosstalk between Wnt and Shh signalling to maintain CSC properties, although more research in that area is currently being carried out to elucidate this.

### 3.4. Targeting Crosstalk of Signalling Pathways in Cancer Stem Cells

Knowing the significant pathways involved with crosstalk in the Shh signalling pathway allows for us to develop treatments accordingly. This knowledge equips us to develop combined treatment strategies that will not be resisted due to this crosstalk, as shown in Figure 2. Research into the Wnt, Hh, and Notch signalling pathways has shown that these critical pathways interact to regulate CSCs [112]. Initially, efforts to target these pathways focused on them individually, leading to issues such as drug resistance and increased metastasis [106,113]. Consequently, more recent investigations have delved into the interconnected nature of these pathways, uncovering how they influence each other. MAML1, a Notch pathway co-activator, also activates the Wnt and Hh pathways through transcriptional regulation, while inhibiting GSK-3β can disrupt all three pathways [114,115]. Targeting these intrapathway interactions presents a promising strategy against cancer progression and drug resistance, with molecules like Zic1 [116,117], SIRT1 [118], and NRF2 [119] emerging as potential targets for future chemotherapy. Furthermore, long noncoding RNAs (LncRNAs) like HOTAIR, MALAT1, MEG3, NEAT1, GAS5, and others have been identified as regulators of the Wnt, Hh, and Notch pathways, both transcriptionally and post-transcriptionally [120,121,122]. These LncRNAs offer potential as diagnostic and prognostic markers, as well as therapeutic targets, due to their specific localisation and genomic proximity. Techniques such as siRNA, ASOs, CRISPR/Cas9, and others could target these epigenetically active LncRNAs, offering a novel approach to cancer treatment [123]. Tumour-suppressive LncRNAs, including MEG3 and GAS5, hold promise as therapeutic agents. However, the clinical application of LncRNAs in cancer therapy faces challenges related to their stability and specificity. Developing new vectors or nanoparticle-based delivery systems could enhance the therapeutic potential of these tumour-suppressive LncRNAs, overcoming existing obstacles to their clinical use.

## 4. Regulation of the Shh Pathway in CSCs

**Figure 3:**
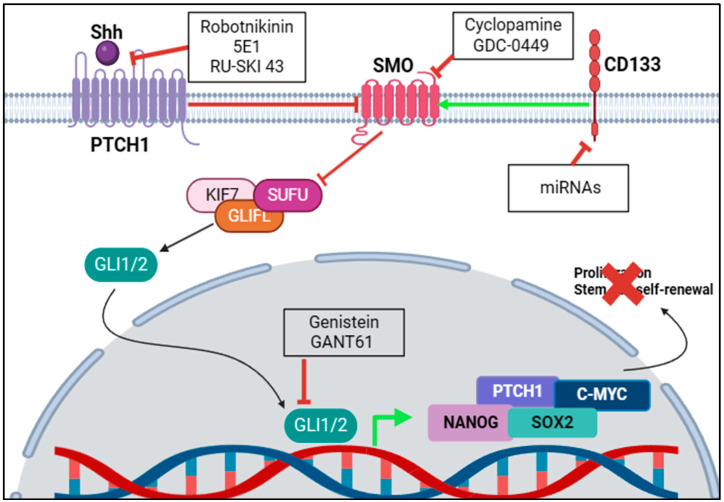
Targeting the Shh signalling pathway regulators. Inhibition of a signalling pathway requires identification of the pathway’s key regulators. Some strategies involve targeting SHH ligand, the most upstream regulator of Shh signalling, which have some positive results but are not yet clinically approved. The majority of targeting strategies are aimed at SMO, as it is the first oncogenic protein in the signalling process, showing the most success in inhibiting Shh signalling with many successful clinical trials and some FDA-approved compounds. Other strategies target GLI1/2, the key transcription factor in Shh signalling, showing some success in inhibition and moving into phase 2 of clinical trials. Newer studies are looking at inhibiting CD133, a surface protein that enhances Shh signalling, with microRNAs, although direct inhibition has not yet been achieved. The red X indicates Proliferation and stem-cell self-renewal are being inhibited.

Knowing the key regulators of the Shh signalling pathways allows for the design of specific inhibitors that can target the upstream and downstream mediators of the pathway shown in Figure 3 and Table 1.

### 4.1. Targeting Shh Ligand

The Shh ligand is the primary upstream regulator of Shh signalling and is the most common ligand that is expressed in cells to initiate Shh signalling. There are very few studies that target the Shh ligand, possibly due to it being an upstream regulator where inhibition can be bypassed through downstream mutations. However, there are some compounds being investigated in vitro that inhibit both the expression of Shh ligand and its ability to bind to PTCH1 preventing Shh signalling.

Recent studies have investigated the monoclonal antibody known as 5E1, which binds to the Shh ligand, preventing its interaction with PTCH1 [124,125]. This antibody has demonstrated efficacy in suppressing the growth of patient-derived oesophageal xenograft models when used in conjunction with radiation therapy [126]. Additionally, in cervical cancer xenografts, 5E1 has been observed to enhance the effects of platinum-based chemotherapy administered concurrently with radiation therapy, although it had no effect on tumorigenesis on its own [113]. Furthermore, in models of gastric cancer, 5E1 has been found to augment the effectiveness of platinum-based therapy [127]. In mouse models of breast cancer, 5E1 has exhibited the ability to reduce tumour size as well as liver and pancreatic metastases [128]. Moreover, when tested as a monotherapy in a mouse model of medulloblastoma, 5E1 effectively suppressed tumour growth and extended survival time [129].

Another molecule known as Robotnikinin also targets the Shh ligand, binding antagonistically and inhibiting its ability to bind to PTCH1, consequently reducing GLI1 activity [130]. Robotnikinin was discovered through screening many molecules that suppress the Shh pathway [131]. However, despite its potential, Robotnikinin has not yet demonstrated any antitumor effects in preclinical models [131].

The synthesis of Shh in the cell requires an enzyme known as Shhat. This enzyme catalyses the N-terminal palmitoylation of the Shh ligand, an essential process that maintains the strength of Shh signalling. RU-SKI 43 is a compound that was discovered through a screening process for Shhat inhibitors, and it was found to effectively suppress both Shh production and signalling of Shh ligand, through inhibition of Shhat [5,73]. Its promise as a therapeutic was evident with it reducing the proliferation and anchorage-independent growth of breast cancer cells [132]. These studies all show the potential of targeting the CSC regulator Shh to inhibit CSC activity and tumorigenesis in various types of cancers.

### 4.2. Targeting Messenger Protein SMO

SMO is a downstream regulator of Shh signalling and is the key signalling protein required for the activation of GLI1/2. Studies investigating SMO as a regulator found that it was often upregulated while PTCH1 was downregulated in multiple myeloma (MM) pluripotent cells, allowing for SMO to act constitutively and maintain pluripotency [133]. Others found a mutation in the C-terminal lysine (K575M) that allows for SMO to act independently of PTCH1 as it is unable to bind to and inhibit SMO, leading to uncontrolled signalling and expression of stem cell renewal genes in hepatocellular carcinoma cells (HCC) [134,135].

Inhibition of SMO with both cyclopamine and siRNA showed reduced clonogenicity and pluripotency in MM cells, inducing significant cell differentiation and decreasing the MM CSC population [133]. Treatment of thyroid CSCs with cyclopamine and GANT61 also led to decreased stem cell populations as well as *SOX2* expression [77]. Inhibition of SMO with GDC-0449 in HCC and colon cancer led to decreased tumorigenesis [134] and SMO knockdown in pancreatic cancer caused a decrease in CSC population as well as the expression of NANOG, a stem cell self-renewal protein [136]. This evidence highlights SMO as a potential target for combined chemotherapy and/or radiation therapies that could potentially bypass CSC induced resistance to treatment.

### 4.3. Targeting GLI1/2

GLI1/2 is another key mediator of Shh signalling; it is the transcription factor responsible for the expression of the stem cell self-renewal genes such as *NANOG*, *OCT3/4*, and *SOX2*. It is also one of the downstream regulators that allow for crosstalk between Shh, Wnt, and PI3K signalling. This emphasises it as a significant target for combination therapies. Inhibiting upstream targets in Shh signalling would not alleviate the effects of the Wnt and PI3K crosstalk, which would allow for the Shh target genes to be expressed even though Shh signalling is inhibited.

Ciclesonide, an FDA-approved corticosteroid for the treatment of asthma, has been tested in lung cancer in the hopes of repurposing therapeutics. It was found that ciclesonide reduced tumorigenesis of lung cells through the inhibition of *GLI1/2* and *SOX2* expression, marking it as a potential therapeutic for lung cancer patients [137].

Genistein, an isoflavone derived from Genista tinctoria, has drawn attention in recent years for its ability to suppress GLI1, particularly within the CSC niche, across various tumour types. It was shown to reduce stemness and the CSC population in prostate and breast cancer cells through the inhibition of GLI1 [138,139]. More recent studies on nasopharyngeal cancer, renal cancer, and gastric cancer also found that genistein suppresses Shh and GLI1 signalling, decreasing the expression of CD44 and other stem cell markers. In addition, genistein was shown to inhibit proliferation and tumour spheroid formation and initiate apoptosis in all three CSC types [140,141,142]. Phase I trials investigating genistein have demonstrated a notably favourable safety profile, prompting the initiation of several phase II trials [131]. While genistein has shown some efficacy in prostate cancers in these trials, its effectiveness in pancreatic cancers has yielded mixed results [143,144].

GANT61 is another GLI1 inhibitor that demonstrates significant efficacy in reducing GLI1/2 DNA binding [145]. A study investigating GANT61 in embryonal and alveolar RMS found that it was able to reduce proliferation in both RMS types through inhibiting GLI1 and initiating cell cycle arrest, bypassing SMO inhibition resistance [146]. Another study investigated both GLI1 and SMO inhibition in prostate CSCs using GANT61 and GDC-0449, respectively. Both compounds led to a decrease in CSC populations; however, GANT-61 induced apoptosis more effectively compared to GDC-0449 [147]. This is possibly because GLI1 is further downstream than SMO while simultaneously being a site of crosstalk between multiple signalling pathways. Inhibition of GLI1 directly possibly had more of an effect than just SMO because the crosstalk signalling was inhibited simultaneously. GANT61 was also shown to reduce oestrogen receptor-positive breast CSC populations through inhibition of GLI1/2 activity and inducing apoptosis in breast CSCs, while also inhibiting mammosphere formation ability [148]. Despite its promising preclinical results, to date, there are no registered clinical trials for GANT61, and studies remain in the preclinical phase.

### 4.4. miRNAs Targeting CD133

MicroRNAs (miRNAs) are small noncoding RNAs that play significant roles in various biological processes, including cancer progression. They can influence cancer development through mechanisms like gene amplification or deletion, abnormal transcriptional regulation, and epigenetic alterations. MiRNAs are also crucial in regulating signalling pathways essential for proper cellular functions [149]. The importance of miRNAs in stem cell regulation was initially identified by Lee, Feinbaum, and Ambros in 1993, with further studies observing their regulatory effects on CD133+ CSCs [150,151]. These miRNAs can act as both tumour promoters and suppressors across different cell types and tissues by modulating signalling pathways [152].

In HCC, the overexpression of EIF5A2 leads to increased CD133 expression and tumour development. The suppression of miR-29b, which acts as a tumour suppressor, is linked to the maintenance of stemness in HCC cells by EIF5A2 [153]. The specific manner in which EIF5A2 influences miRNA-29b regulation is not fully understood; however, it has been documented that factors such as c-MYC, Hh, and NF-κB can inhibit miR-29 expression at the transcriptional level [154]. The miR-30 family plays dual roles in cancer, acting as tumour promoters in pancreatic cancer by enhancing the migratory and invasive capacities of CD133+ cells, while functioning as tumour suppressors in lung, breast, and gastric cancers [155]. This family is known to suppress EMT by targeting vimentin, a process influenced by various pathways including TGF-β, TNF-α, Wnt, Notch, and Hh [156]. In glioblastoma, miR-9 is upregulated in CD133+ cells, leading to chemoresistance by activating the Shh signalling pathway and downregulating the Shh receptor PTCH1 at the post-transcriptional level [61]. This action reduces cell death, while knockdown of GLI1 and MDR1 can enhance cell death induced by TMZ. Notably, CD133 and MDR protein 1 levels increase in recurrent cancers after prolonged chemotherapy [157]. Despite these insights, the direct targeting of CD133 expression by specific miRNAs remains unknown, with most miRNAs showing indirect regulation of CD133 expression.

## 5. Small Molecule Inhibitors

Despite the existence of neutralizing anti-Shh antibodies, alternative potent inhibitors that disrupt the Shh–Ptch interaction have been identified. Stanton et al. documented the moderate Shh inhibitory activity of Robotnikinin, the first small molecular weight Shh inhibitor [130]. The second small molecular weight Shh inhibitor, HL2-m5, was a macrocyclic peptide [158]. More recently, seven additional organic small molecule inhibitors with protein–protein interactions with Shh have been identified [159]. Another small molecule inhibitor of Shh is apatinib, also known as rivoceranib, is recognised as an oral small molecule tyrosine kinase inhibitor [160]. It was repurposed and demonstrated efficacy in suppressing characteristics of gastric CSCs by inhibiting tumoursphere formation and cell proliferation, reducing expression of gastric CSC markers and CD133+ cell count, and inducing apoptosis. It downregulated activation of the Shh pathway, while upregulation of the Shh pathway decreased the inhibitory effects of apatinib on gastric CSCs. Furthermore, in a xenograft model, apatinib treatment significantly retarded tumour growth and hindered gastric CSC characteristics [161].

In a study that examined and compared the impact of three small molecule inhibitors: XAV939 (Wnt pathway), GDC0449 (Hedgehog pathway), and DAPT (Notch pathway). Inhibitors for the Wnt and Notch pathways resulted in smaller and fewer spheroids, while the Hedgehog pathway inhibitor completely halted spheroid formation in an ovarian cancer cell line. Similar effects were observed in other cell lines. Additional Hedgehog pathway inhibitors, including LDE225 and GANT61, yielded comparable outcomes [162]. GANT61 was the initial small molecule inhibitor targeting GLI1 [145]. It has since proven to significantly decrease the proportion of CSCs in triple-negative breast cancer cells [163]. Moreover, the combination of GANT61 with mTOR inhibition has offered a promising strategy for effectively suppressing pancreatic CSCs, thus enhancing the treatment of pancreatic cancer [164].

The natural compound cyclopamine inhibits Shh signalling by targeting SMO. Mounting evidence suggests that cyclopamine exhibits anti-cancer properties across various human cancer types by inhibiting the proliferation of CSCs [165,166]. It has been found to successfully impede the expression of CSC markers in pancreatic cancer cells [167]. Vismodegib (GDC-0449) is a small molecule Shh pathway antagonist that binds to SMO [168]. It suppresses the biological activities of pancreatic CSCs associated with malignancy [169]. Additionally, vismodegib triggers apoptosis in pancreatic CSCs by activating caspase 3 and inducing PARP cleavage. It also exhibits various effects on breast CSCs, such as inhibiting proliferation and invasion, and promoting apoptosis [170]. However, despite its efficacy against basal cell carcinoma, vismodegib’s clinical use is limited due to severe side effects, low selectivity for CSCs, and the emergence of drug resistance [171]. Clinical observations of drug-resistant SMO mutations and aberrant Hh signalling downstream of SMO underscore the need for alternative therapeutic approaches [172]. Research is exploring novel SMO antagonists capable of overcoming drug resistance, along with downstream Hh inhibitors targeting GLI proteins, and the efficacy of combining various targeting agents for effective Hh inhibition [173,174].

## 6. Future Prospective

Three out of the five FDA-approved Shh inhibitors mentioned in this review (Table 2) target the SMO receptor protein, with two of them (sonidegib and vismodegib) having the exact same binding pocket. This is an issue, as a mutation in this binding pocket can lead to resistance to both drugs, leaving only one discussed FDA-approved SMO inhibitor. The research needs to shift towards compounds that target other aspects of Shh signalling. As seen in Table 2, there are attempts to target Shh ligand and GLI1/2; however, no Shh inhibitors have made it to the clinical trials stage, remaining in vitro, while few GLI1/2 inhibitors have begun clinical trials. There are two FDA-approved drugs that target GLI1/2; however, ciclesonide was initially approved for treatment of asthma, and its mechanism of inhibiting GLI1/2/SOX2 expression is unknown [175]. Overcoming resistance to treatment requires a multifaceted approach, including the identification of multiple pathway modulators, understanding resistance mechanisms, and the development of combination therapies that target multiple Shh signalling modulators as well as crosstalk signalling pathways. Strategies such as targeting pathway crosstalk, improving drug delivery systems, and personalizing treatments based on genetic profiling of tumours could enhance the efficacy of Shh pathway-targeted interventions. Emphasising the intricate relationship between the Shh pathway and CSCs will be crucial in advancing our quest for more effective cancer therapies, offering hope for improved patient outcomes. Such approaches hold the promise of more effectively eradicating CSCs and achieving better control over cancer progression.

## 7. Conclusions

The Shh pathway plays a pivotal role in the regulation of CSCs, influencing the progression, prognosis, and therapeutic outcomes across a spectrum of cancers. The activation of the Shh pathway in CSCs is associated with a more aggressive cancer phenotype, poorer prognosis, and reduced responsiveness to conventional therapies, underscoring its clinical significance. Efforts to target the Shh pathway have led to the development of various inhibitors, some of which are currently being evaluated in clinical trials. These therapeutic strategies aim to disrupt the Shh signalling, thereby diminishing the CSC population, limiting tumour growth and resistance, and improving patient outcomes. However, the efficacy of Shh pathway inhibitors has been mixed, with challenges including the development of resistance and the complexity of CSC biology.

Targeting the Shh pathway in CSCs represents a promising avenue for cancer treatment. Future research on the Shh pathway in CSCs is still needed for unveiling novel therapeutic targets to overcome drug resistance, through both combination therapy and targeting cell signalling crosstalk, as well as enhancing our understanding of cancer biology.

## Figures and Tables

**Table 1:** Key regulators of Shh signalling and their mechanisms of action.

Regulator	Role in Signalling
HHAT	Essential for Shh palmitoylation, stabilizing the ligand for secretion.
DISP1	Required for long-range secretion of Shh to target cells.
Shh Ligand	Signal activator, initiates the Shh cascade by inhibiting PTCH1.
PTCH1	In absence of Shh ligand, it prevents activation of Shh signalling by inhibiting SMO activity.
SMO	Inhibits SUFU activity when Shh ligand is present, which leads to GLI1/2 expression and signalling.
SUFU	When Shh is not present, prevents expression of GLI1/2 and produces GLIRepressor. When Shh is present, it gets inhibited, and GLI1/2 is produced.
GLI1/2	Moves into nucleus and initiates transcription of target genes *NANOG*, *SOX2*.
GLIRepressor	Moves into nucleus and inhibits expression of GLI1/2 target genes.

All regulators summarised in this table were referenced earlier in this paper. Not all known regulators of Shh were discussed and we acknowledge that this table does not represent all regulators.

**Table 2:** Some of the current and developing inhibitors of the Shh signalling pathway.

Inhibitor	Target	Mechanism of Action	Clinical Trial Status	Trial Number (s)
Sonidegib	SMO	SMO receptor inhibitor	FDA-approved	
Vismodegib	SMO	SMO receptor inhibitor	FDA-approved	
ATO	GLI1	Inhibits GLI1 activity	FDA-approved	
Glasdegib	SMO	SMO receptor inhibitor	FDA-approved	
Ciclesonide	SMO/GLI1/2	Exact mechanism is unknown	FDA-approved	
ROBOTNIKININ	Shh	Shh ligand inhibitor	No trials to date *	N/A
5E1	Shh	Shh ligand inhibitor	No trials to date *	N/A
RU-SKI 43	Shh	Shhat enzyme inhibitor, preventing Shh ligand synthesis	No trials to date *	N/A
Saridegib	SMO	SMO receptor inhibitor	Phase 2	NCT01310816, NCT01130142
Talidegib	SMO	SMO receptor inhibitor	Phase 2	NCT05199584, NCT02530437, NCT01722292
LEQ506	SMO	SMO receptor inhibitor	Phase 1	NCT01106508
Cyclopamine	SMO	SMO receptor inhibitor	No trials to date *	N/A
Rivoceranib	SMO/GLI1/2	Exact mechanism is unknown	Phase 3	NCT03042611, NCT04639180
Genistein	GLI1/2	Decreases GLI1 activity, specifics unknown	Phase 2 and 3	NCT01985763, NCT01126879, NCT00584532
GANT61	GLI1/2	Inhibits GLI1/2 DNA binding ability	No trials to date *	N/A
Pirfenidone	GLI2	Destabilises GLI2 protein to prevent activity	Phase 2	NCT06142318, NCT05801133
miRNAs	CD133	Inhibits expression of CD133	No trials to date *	N/A

* At the time of writing this review, no trials were found on clinicaltrials.gov. We acknowledge that this table does not discuss all known Shh inhibitors.

## References

[1] Echelard Y., Epstein D.J., St-Jacques B., Shen L., Mohler J., McMahon J.A., McMahon A.P. (1993). Sonic hedgehog, a member of a family of putative signaling molecules, is implicated in the regulation of CNS polarity. Cell.

[2] Li X., Li Y., Li S., Li H., Yang C., Lin J. (2021). The role of Shh signalling pathway in central nervous system development and related diseases. Cell Biochem. Funct..

[3] Yang C., Qi Y., Sun Z. (2021). The Role of Sonic Hedgehog Pathway in the Development of the Central Nervous System and Aging-Related Neurodegenerative Diseases. Front. Mol. Biosci..

[4] Adelian S., Ahadi A.M., Ayat H., Teimori H. (2020). Enhanced recombinant C-terminal domain of gli2 gene expression can improve wound healing through promoting cdc25b and N-Myc genes expression. Gene Rep..

[5] Petrova E., Rios-Esteves J., Ouerfelli O., Glickman J.F., Resh M.D. (2013). Inhibitors of Hedgehog acyltransferase block Sonic Hedgehog signaling. Nat. Chem. Biol..

[6] Rohatgi R., Milenkovic L., Scott M.P. (2007). Patched1 regulates hedgehog signaling at the primary cilium. Science.

[7] Pietrobono S., Gagliardi S., Stecca B. (2019). Non-canonical Hedgehog Signaling Pathway in Cancer: Activation of GLI Transcription Factors Beyond Smoothened. Front. Genet..

[8] Huangfu D., Liu A., Rakeman A.S., Murcia N.S., Niswander L., Anderson K.V. (2003). Hedgehog signalling in the mouse requires intraflagellar transport proteins. Nature.

[9] Bangs F., Anderson K.V. (2017). Primary Cilia and Mammalian Hedgehog Signaling. Cold Spring Harb. Perspect. Biol..

[10] Goetz S.C., Anderson K.V. (2010). The primary cilium: A signalling centre during vertebrate development. Nat. Rev. Genet..

[11] Carballo G.B., Honorato J.R., de Lopes G.P.F., Spohr T. (2018). A highlight on Sonic hedgehog pathway. Cell Commun. Signal..

[12] Niewiadomski P., Kong J.H., Ahrends R., Ma Y., Humke E.W., Khan S., Teruel M.N., Novitch B.G., Rohatgi R. (2014). Gli protein activity is controlled by multisite phosphorylation in vertebrate Hedgehog signaling. Cell Rep..

[13] Kogerman P., Grimm T., Kogerman L., Krause D., Unden A.B., Sandstedt B., Toftgard R., Zaphiropoulos P.G. (1999). Mammalian suppressor-of-fused modulates nuclear-cytoplasmic shuttling of Gli-1. Nat. Cell Biol..

[14] Ingham P.W., McMahon A.P. (2009). Hedgehog signalling: Kif7 is not that fishy after all. Curr. Biol..

[15] Pan Y., Bai C.B., Joyner A.L., Wang B. (2006). Sonic hedgehog signaling regulates Gli2 transcriptional activity by suppressing its processing and degradation. Mol. Cell. Biol..

[16] Huntzicker E.G., Estay I.S., Zhen H., Lokteva L.A., Jackson P.K., Oro A.E. (2006). Dual degradation signals control Gli protein stability and tumor formation. Genes Dev..

[17] Chen Y., Sasai N., Ma G., Yue T., Jia J., Briscoe J., Jiang J. (2011). Sonic Hedgehog dependent phosphorylation by CK1alpha and GRK2 is required for ciliary accumulation and activation of smoothened. PLoS Biol..

[18] Gupta S., Takebe N., Lorusso P. (2010). Targeting the Hedgehog pathway in cancer. Ther. Adv. Med. Oncol..

[19] Ikram M.S., Neill G.W., Regl G., Eichberger T., Frischauf A.M., Aberger F., Quinn A., Philpott M. (2004). GLI2 is expressed in normal human epidermis and BCC and induces GLI1 expression by binding to its promoter. J. Investig. Dermatol..

[20] Dai P., Akimaru H., Tanaka Y., Maekawa T., Nakafuku M., Ishii S. (1999). Sonic Hedgehog-induced activation of the Gli1 promoter is mediated by GLI3. J. Biol. Chem..

[21] Brennan D., Chen X., Cheng L., Mahoney M., Riobo N.A. (2012). Noncanonical Hedgehog signaling. Vitam. Horm..

[22] Jenkins D. (2009). Hedgehog signalling: Emerging evidence for non-canonical pathways. Cell Signal..

[23] Copp A.J., Greene N.D. (2010). Genetics and development of neural tube defects. J. Pathol..

[24] Murdoch J.N., Copp A.J., Teratology M. (2010). The relationship between sonic Hedgehog signaling, cilia, and neural tube defects. Birth Defects Res. A Clin. Mol. Teratol..

[25] Hong M., Krauss R.S. (2018). Modeling the complex etiology of holoprosencephaly in mice. Am. J. Med. Genet. C Semin. Med. Genet..

[26] Hammond N.L., Brookes K.J., Dixon M.J. (2018). Ectopic Hedgehog Signaling Causes Cleft Palate and Defective Osteogenesis. J. Dent. Res..

[27] Ehlen H.W., Buelens L.A., Vortkamp A. (2006). Hedgehog signaling in skeletal development. Birth Defects Res. C Embryo Today.

[28] Varnat F., Duquet A., Malerba M., Zbinden M., Mas C., Gervaz P., Ruiz i Altaba A. (2009). Human colon cancer epithelial cells harbour active HEDGEHOG-GLI signalling that is essential for tumour growth, recurrence, metastasis and stem cell survival and expansion. EMBO Mol. Med..

[29] Fukaya M., Isohata N., Ohta H., Aoyagi K., Ochiya T., Saeki N., Yanagihara K., Nakanishi Y., Taniguchi H., Sakamoto H. (2006). Hedgehog signal activation in gastric pit cell and in diffuse-type gastric cancer. Gastroenterology.

[30] Pasca di Magliano M., Sekine S., Ermilov A., Ferris J., Dlugosz A.A., Hebrok M. (2006). Hedgehog/Ras interactions regulate early stages of pancreatic cancer. Genes Dev..

[31] Karhadkar S.S., Bova G.S., Abdallah N., Dhara S., Gardner D., Maitra A., Isaacs J.T., Berman D.M., Beachy P.A. (2004). Hedgehog signalling in prostate regeneration, neoplasia and metastasis. Nature.

[32] Hanna A., Shevde L.A. (2016). Hedgehog signaling: Modulation of cancer properies and tumor mircroenvironment. Mol. Cancer.

[33] Cochrane C.R., Szczepny A., Watkins D.N., Cain J.E. (2015). Hedgehog Signaling in the Maintenance of Cancer Stem Cells. Cancers.

[34] Amakye D., Jagani Z., Dorsch M. (2013). Unraveling the therapeutic potential of the Hedgehog pathway in cancer. Nat. Med..

[35] Coletta R.D., Yeudall W.A., Salo T. (2020). Grand Challenges in Oral Cancers. Front. Oral Health.

[36] Baniebrahimi G., Mir F., Khanmohammadi R. (2020). Cancer stem cells and oral cancer: Insights into molecular mechanisms and therapeutic approaches. Cancer Cell Int..

[37] O’Brien C.A., Kreso A., Jamieson C.H. (2010). Cancer stem cells and self-renewal. Clin. Cancer Res..

[38] Chen K., Huang Y.H., Chen J.L. (2013). Understanding and targeting cancer stem cells: Therapeutic implications and challenges. Acta Pharmacol. Sin..

[39] Chen D., Wang C.Y. (2019). Targeting cancer stem cells in squamous cell carcinoma. Precis. Clin. Med..

[40] Zhang X., Powell K., Li L. (2020). Breast Cancer Stem Cells: Biomarkers, Identification and Isolation Methods, Regulating Mechanisms, Cellular Origin, and Beyond. Cancers.

[41] Celia-Terrassa T., Jolly M.K. (2020). Cancer Stem Cells and Epithelial-to-Mesenchymal Transition in Cancer Metastasis. Cold Spring Harb. Perspect. Med..

[42] Chambers A.F., Groom A.C., MacDonald I.C. (2002). Dissemination and growth of cancer cells in metastatic sites. Nat. Rev. Cancer.

[43] Celia-Terrassa T., Kang Y. (2016). Distinctive properties of metastasis-initiating cells. Genes Dev..

[44] Esposito M., Mondal N., Greco T.M., Wei Y., Spadazzi C., Lin S.C., Zheng H., Cheung C., Magnani J.L., Lin S.H. (2019). Bone vascular niche E-selectin induces mesenchymal-epithelial transition and Wnt activation in cancer cells to promote bone metastasis. Nat. Cell Biol..

[45] Pastushenko I., Blanpain C. (2019). EMT Transition States during Tumor Progression and Metastasis. Trends Cell Biol..

[46] Krishnaswamy S., Zivanovic N., Sharma R., Pe’er D., Bodenmiller B. (2018). Learning time-varying information flow from single-cell epithelial to mesenchymal transition data. PLoS ONE.

[47] van Dijk D., Sharma R., Nainys J., Yim K., Kathail P., Carr A.J., Burdziak C., Moon K.R., Chaffer C.L., Pattabiraman D. (2018). Recovering Gene Interactions from Single-Cell Data Using Data Diffusion. Cell.

[48] Karacosta L.G., Anchang B., Ignatiadis N., Kimmey S.C., Benson J.A., Shrager J.B., Tibshirani R., Bendall S.C., Plevritis S.K. (2019). Mapping lung cancer epithelial-mesenchymal transition states and trajectories with single-cell resolution. Nat. Commun..

[49] Malta T.M., Sokolov A., Gentles A.J., Burzykowski T., Poisson L., Weinstein J.N., Kaminska B., Huelsken J., Omberg L., Gevaert O. (2018). Machine Learning Identifies Stemness Features Associated with Oncogenic Dedifferentiation. Cell.

[50] George J.T., Jolly M.K., Xu S., Somarelli J.A., Levine H. (2017). Survival Outcomes in Cancer Patients Predicted by a Partial EMT Gene Expression Scoring Metric. Cancer Res..

[51] Ko Y.C., Choi H.S., Liu R., Lee D.S. (2021). Physalin A, 13,14-Seco-16, 24-Cyclo-Steroid, Inhibits Stemness of Breast Cancer Cells by Regulation of Hedgehog Signaling Pathway and Yes-Associated Protein 1 (YAP1). Int. J. Mol. Sci..

[52] Gkountela S., Castro-Giner F., Szczerba B.M., Vetter M., Landin J., Scherrer R., Krol I., Scheidmann M.C., Beisel C., Stirnimann C.U. (2019). Circulating Tumor Cell Clustering Shapes DNA Methylation to Enable Metastasis Seeding. Cell.

[53] Malladi S., Macalinao D.G., Jin X., He L., Basnet H., Zou Y., de Stanchina E., Massague J. (2016). Metastatic Latency and Immune Evasion through Autocrine Inhibition of WNT. Cell.

[54] Guo W., Keckesova Z., Donaher J.L., Shibue T., Tischler V., Reinhardt F., Itzkovitz S., Noske A., Zurrer-Hardi U., Bell G. (2012). Slug and Sox9 cooperatively determine the mammary stem cell state. Cell.

[55] Celia-Terrassa T., Liu D.D., Choudhury A., Hang X., Wei Y., Zamalloa J., Alfaro-Aco R., Chakrabarti R., Jiang Y.Z., Koh B.I. (2017). Normal and cancerous mammary stem cells evade interferon-induced constraint through the miR-199a-LCOR axis. Nat. Cell Biol..

[56] Ma C., Zhao J.Z., Lin R.T., Zhou L., Chen Y.N., Yu L.J., Shi T.Y., Wang M., Liu M.M., Liu Y.R. (2018). Combined overexpression of cadherin 6, cadherin 11 and cluster of differentiation 44 is associated with lymph node metastasis and poor prognosis in oral squamous cell carcinoma. Oncol. Lett..

[57] Shin K.H., Kim R.H. (2018). An Updated Review of Oral Cancer Stem Cells and Their Stemness Regulation. Crit. Rev. Oncog..

[58] Yan Y., Zuo X., Wei D. (2015). Concise Review: Emerging Role of CD44 in Cancer Stem Cells: A Promising Biomarker and Therapeutic Target. Stem Cells Transl. Med..

[59] Wang L., Zuo X., Xie K., Wei D. (2018). The Role of CD44 and Cancer Stem Cells. Methods Mol. Biol..

[60] Chen Y., Sun W., He R., Zhang F., Wang H., Li P., Shao R.G., Xu X. (2017). Lidamycin decreases CD133 expression in hepatocellular carcinoma via the Notch signaling pathway. Oncol. Lett..

[61] Aghajani M., Mansoori B., Mohammadi A., Asadzadeh Z., Baradaran B. (2019). New emerging roles of CD133 in cancer stem cell: Signaling pathway and miRNA regulation. J. Cell. Physiol..

[62] Cierpikowski P., Lis-Nawara A., Bar J. (2021). SHH Expression Is Significantly Associated With Cancer Stem Cell Markers in Oral Squamous Cell Carcinoma. Anticancer Res..

[63] Chen J.-H., Zhai E.-T., Chen S.-L., Wu H., Wu K.-M., Zhang X.-H., Chen C.-Q., Cai S.-R., He Y.-L. (2016). CD44, Sonic Hedgehog, and Gli1 Expression Are Prognostic Biomarkers in Gastric Cancer Patients after Radical Resection. Gastroenterol. Res. Pract..

[64] Jeng K.S., Sheen I.S., Jeng W.J., Yu M.C., Hsiau H.I., Chang F.Y., Tsai H.H. (2013). Activation of the sonic hedgehog signaling pathway occurs in the CD133 positive cells of mouse liver cancer Hepa 1-6 cells. Onco Targets Ther..

[65] Ulasov I.V., Nandi S., Dey M., Sonabend A.M., Lesniak M.S. (2011). Inhibition of Sonic hedgehog and Notch pathways enhances sensitivity of CD133(+) glioma stem cells to temozolomide therapy. Mol. Med..

[66] Taipale J., Beachy P.A. (2001). The Hedgehog and Wnt signalling pathways in cancer. Nature.

[67] Skoda A.M., Simovic D., Karin V., Kardum V., Vranic S., Serman L. (2018). The role of the Hedgehog signaling pathway in cancer: A comprehensive review. Bosn. J. Basic Med. Sci..

[68] Reifenberger J., Wolter M., Knobbe C.B., Kohler B., Schonicke A., Scharwachter C., Kumar K., Blaschke B., Ruzicka T., Reifenberger G. (2005). Somatic mutations in the PTCH, SMOH, SUFUH and TP53 genes in sporadic basal cell carcinomas. Br. J. Dermatol..

[69] Ingham P.W., McMahon A.P. (2001). Hedgehog signaling in animal development: Paradigms and principles. Genes Dev..

[70] Szkandera J., Kiesslich T., Haybaeck J., Gerger A., Pichler M. (2013). Hedgehog signaling pathway in ovarian cancer. Int. J. Mol. Sci..

[71] Etheridge L.A., Crawford T.Q., Zhang S., Roelink H. (2010). Evidence for a role of vertebrate Disp1 in long-range Shh signaling. Development.

[72] Chen M.H., Li Y.J., Kawakami T., Xu S.M., Chuang P.T. (2004). Palmitoylation is required for the production of a soluble multimeric Hedgehog protein complex and long-range signaling in vertebrates. Genes Dev..

[73] Buglino J.A., Resh M.D. (2008). Hhat is a palmitoylacyltransferase with specificity for N-palmitoylation of Sonic Hedgehog. J. Biol. Chem..

[74] Nicolis S.K. (2007). Cancer stem cells and “stemness” genes in neuro-oncology. Neurobiol. Dis..

[75] Zhao C., Chen A., Jamieson C.H., Fereshteh M., Abrahamsson A., Blum J., Kwon H.Y., Kim J., Chute J.P., Rizzieri D. (2009). Hedgehog signalling is essential for maintenance of cancer stem cells in myeloid leukaemia. Nature.

[76] Xu X., Lu Y., Li Y., Prinz R.A. (2017). Sonic Hedgehog Signaling in Thyroid Cancer. Front. Endocrinol..

[77] Lu Y., Zhu Y., Deng S., Chen Y., Li W., Sun J., Xu X. (2021). Targeting the Sonic Hedgehog Pathway to Suppress the Expression of the Cancer Stem Cell (CSC)-Related Transcription Factors and CSC-Driven Thyroid Tumor Growth. Cancers.

[78] Heiden K.B., Williamson A.J., Doscas M.E., Ye J., Wang Y., Liu D., Xing M., Prinz R.A., Xu X. (2014). The sonic hedgehog signaling pathway maintains the cancer stem cell self-renewal of anaplastic thyroid cancer by inducing snail expression. J. Clin. Endocrinol. Metab..

[79] Carina V., Zito G., Pizzolanti G., Richiusa P., Criscimanna A., Rodolico V., Tomasello L., Pitrone M., Arancio W., Giordano C. (2013). Multiple pluripotent stem cell markers in human anaplastic thyroid cancer: The putative upstream role of SOX2. Thyroid.

[80] Satheesha S., Manzella G., Bovay A., Casanova E.A., Bode P.K., Belle R., Feuchtgruber S., Jaaks P., Dogan N., Koscielniak E. (2016). Targeting hedgehog signaling reduces self-renewal in embryonal rhabdomyosarcoma. Oncogene.

[81] Zhu R., Gires O., Zhu L., Liu J., Li J., Yang H., Ju G., Huang J., Ge W., Chen Y. (2019). TSPAN8 promotes cancer cell stemness via activation of sonic Hedgehog signaling. Nat. Commun..

[82] Chen G., Yin S., Zeng H., Li H., Wan X. (2022). Regulation of Embryonic Stem Cell Self-Renewal. Life.

[83] Li W., Lee M.R., Kim T., Kim Y.W., Cho M.Y. (2018). Activated STAT3 may participate in tumor progression through increasing CD133/survivin expression in early stage of colon cancer. Biochem. Biophys. Res. Commun..

[84] Jang J.W., Song Y., Kim S.H., Kim J.S., Kim K.M., Choi E.K., Kim J., Seo H.R. (2017). CD133 confers cancer stem-like cell properties by stabilizing EGFR-AKT signaling in hepatocellular carcinoma. Cancer Lett..

[85] Oskarsson T., Batlle E., Massague J. (2014). Metastatic stem cells: Sources, niches, and vital pathways. Cell Stem Cell.

[86] Geyer N., Gerling M. (2021). Hedgehog Signaling in Colorectal Cancer: All in the Stroma?. Int. J. Mol. Sci..

[87] Abdelmaksoud N.M., Abulsoud A.I., Doghish A.S., Abdelghany T.M. (2023). From resistance to resilience: Uncovering chemotherapeutic resistance mechanisms; insights from established models. Biochim. Biophys. Acta Rev. Cancer.

[88] Fan F., Wang R., Boulbes D.R., Zhang H., Watowich S.S., Xia L., Ye X., Bhattacharya R., Ellis L.M. (2018). Macrophage conditioned medium promotes colorectal cancer stem cell phenotype via the hedgehog signaling pathway. PLoS ONE.

[89] Jinushi M., Komohara Y. (2015). Tumor-associated macrophages as an emerging target against tumors: Creating a new path from bench to bedside. Biochim. Biophys. Acta.

[90] Yang S.H., Sharrocks A.D., Whitmarsh A.J. (2013). MAP kinase signalling cascades and transcriptional regulation. Gene.

[91] Park J.I. (2023). MAPK-ERK Pathway. Int. J. Mol. Sci..

[92] Burotto M., Chiou V.L., Lee J.M., Kohn E.C. (2014). The MAPK pathway across different malignancies: A new perspective. Cancer.

[93] Rovida E., Stecca B. (2015). Mitogen-activated protein kinases and Hedgehog-GLI signaling in cancer: A crosstalk providing therapeutic opportunities?. Semin. Cancer Biol..

[94] Riobo N.A., Haines G.M., Emerson C.P. (2006). Protein kinase C-delta and mitogen-activated protein/extracellular signal-regulated kinase-1 control GLI activation in hedgehog signaling. Cancer Res..

[95] Whisenant T.C., Ho D.T., Benz R.W., Rogers J.S., Kaake R.M., Gordon E.A., Huang L., Baldi P., Bardwell L. (2010). Computational prediction and experimental verification of new MAP kinase docking sites and substrates including Gli transcription factors. PLoS Comput. Biol..

[96] Ji Z., Mei F.C., Xie J., Cheng X. (2007). Oncogenic KRAS activates hedgehog signaling pathway in pancreatic cancer cells. J. Biol. Chem..

[97] Mazumdar T., Devecchio J., Agyeman A., Shi T., Houghton J.A. (2011). Blocking Hedgehog survival signaling at the level of the GLI genes induces DNA damage and extensive cell death in human colon carcinoma cells. Cancer Res..

[98] Mazumdar T., DeVecchio J., Agyeman A., Shi T., Houghton J.A. (2011). The GLI genes as the molecular switch in disrupting Hedgehog signaling in colon cancer. Oncotarget.

[99] Wei L., Xu Z. (2011). Cross-signaling among phosphinositide-3 kinase, mitogen-activated protein kinase and sonic hedgehog pathways exists in esophageal cancer. Int. J. Cancer.

[100] Ebrahimi N., Afshinpour M., Fakhr S.S., Kalkhoran P.G., Shadman-Manesh V., Adelian S., Beiranvand S., Rezaei-Tazangi F., Khorram R., Hamblin M.R. (2023). Cancer stem cells in colorectal cancer: Signaling pathways involved in stemness and therapy resistance. Crit. Rev. Oncol. Hematol..

[101] Dienstmann R., Rodon J., Serra V., Tabernero J. (2014). Picking the point of inhibition: A comparative review of PI3K/AKT/mTOR pathway inhibitors. Mol. Cancer Ther..

[102] Douville J., Beaulieu R., Balicki D. (2009). ALDH1 as a functional marker of cancer stem and progenitor cells. Stem Cells Dev..

[103] Mohamed S.Y., Kaf R.M., Ahmed M.M., Elwan A., Ashour H.R., Ibrahim A. (2019). The Prognostic Value of Cancer Stem Cell Markers (Notch1, ALDH1, and CD44) in Primary Colorectal Carcinoma. J. Gastrointest. Cancer.

[104] Agrawal K., Chauhan S., Kumar D. (2023). Expression analysis and regulation of GLI and its correlation with stemness and metabolic alteration in human brain tumor. 3 Biotech.

[105] Park J.H., Kim Y.H., Shim S., Kim A., Jang H., Lee S.J., Park S., Seo S., Jang W.I., Lee S.B. (2021). Radiation-Activated PI3K/AKT Pathway Promotes the Induction of Cancer Stem-Like Cells via the Upregulation of SOX2 in Colorectal Cancer. Cells.

[106] Zhou J., Zhu G., Huang J., Li L., Du Y., Gao Y., Wu D., Wang X., Hsieh J.T., He D. (2016). Non-canonical GLI1/2 activation by PI3K/AKT signaling in renal cell carcinoma: A novel potential therapeutic target. Cancer Lett..

[107] Zhou C., Du J., Zhao L., Liu W., Zhao T., Liang H., Fang P., Zhang K., Zeng H. (2021). GLI1 reduces drug sensitivity by regulating cell cycle through PI3K/AKT/GSK3/CDK pathway in acute myeloid leukemia. Cell Death Dis..

[108] Katoh M. (2017). Canonical and non-canonical WNT signaling in cancer stem cells and their niches: Cellular heterogeneity, omics reprogramming, targeted therapy and tumor plasticity (Review). Int. J. Oncol..

[109] Valenti G., Quinn H.M., Heynen G., Lan L., Holland J.D., Vogel R., Wulf-Goldenberg A., Birchmeier W. (2017). Cancer Stem Cells Regulate Cancer-Associated Fibroblasts via Activation of Hedgehog Signaling in Mammary Gland Tumors. Cancer Res..

[110] Luo W., Rodriguez M., Valdez J.M., Zhu X., Tan K., Li D., Siwko S., Xin L., Liu M. (2013). Lgr4 is a key regulator of prostate development and prostate stem cell differentiation. Stem Cells.

[111] Wang Y., Dong J., Li D., Lai L., Siwko S., Li Y., Liu M. (2013). Lgr4 regulates mammary gland development and stem cell activity through the pluripotency transcription factor Sox2. Stem Cells.

[112] Bhal S., Kundu C.N. (2023). Targeting crosstalk of signaling pathways in cancer stem cells: A promising approach for development of novel anti-cancer therapeutics. Med. Oncol..

[113] Chaudary N., Pintilie M., Hedley D., Hill R.P., Milosevic M., Mackay H. (2017). Hedgehog inhibition enhances efficacy of radiation and cisplatin in orthotopic cervical cancer xenografts. Br. J. Cancer.

[114] Chatterjee S., Sil P.C. (2019). Targeting the crosstalks of Wnt pathway with Hedgehog and Notch for cancer therapy. Pharmacol. Res..

[115] Quaranta R., Pelullo M., Zema S., Nardozza F., Checquolo S., Lauer D.M., Bufalieri F., Palermo R., Felli M.P., Vacca A. (2017). Maml1 acts cooperatively with Gli proteins to regulate sonic hedgehog signaling pathway. Cell Death Dis..

[116] Paluszczak J., Wisniewska D., Kostrzewska-Poczekaj M., Kiwerska K., Grenman R., Mielcarek-Kuchta D., Jarmuz-Szymczak M. (2017). Prognostic significance of the methylation of Wnt pathway antagonists-CXXC4, DACT2, and the inhibitors of sonic hedgehog signaling-ZIC1, ZIC4, and HHIP in head and neck squamous cell carcinomas. Clin. Oral Investig..

[117] Ge Q., Hu Y., He J., Chen F., Wu L., Tu X., Qi Y., Zhang Z., Xue M., Chen S. (2020). Zic1 suppresses gastric cancer metastasis by regulating Wnt/beta-catenin signaling and epithelial-mesenchymal transition. FASEB J..

[118] Wu Q., Wang Y., Qian M., Qiao Y., Zou S., Chen C., Zhang X., Chen Y., Zhao Y., Zhu G. (2017). Sirt1 suppresses Wnt/betaCatenin signaling in liver cancer cells by targeting betaCatenin in a PKAalpha-dependent manner. Cell Signal..

[119] Leung H.W., Lau E.Y.T., Leung C.O.N., Lei M.M.L., Mok E.H.K., Ma V.W.S., Cho W.C.S., Ng I.O.L., Yun J.P., Cai S.H. (2020). NRF2/SHH signaling cascade promotes tumor-initiating cell lineage and drug resistance in hepatocellular carcinoma. Cancer Lett..

[120] Fu S., Wang Y., Li H., Chen L., Liu Q. (2020). Regulatory Networks of LncRNA MALAT-1 in Cancer. Cancer Manag. Res..

[121] Guo F., Cao Z., Guo H., Li S. (2018). The action mechanism of lncRNA-HOTAIR on the drug resistance of non-small cell lung cancer by regulating Wnt signaling pathway. Exp. Ther. Med..

[122] Qian C.S., Li L.J., Huang H.W., Yang H.F., Wu D.P. (2020). MYC-regulated lncRNA NEAT1 promotes B cell proliferation and lymphomagenesis via the miR-34b-5p-GLI1 pathway in diffuse large B-cell lymphoma. Cancer Cell Int..

[123] Zhen S., Li X. (2019). Application of CRISPR-Cas9 for Long Noncoding RNA Genes in Cancer Research. Hum. Gene Ther..

[124] Ericson J., Morton S., Kawakami A., Roelink H., Jessell T.M. (1996). Two critical periods of Sonic Hedgehog signaling required for the specification of motor neuron identity. Cell.

[125] Maun H.R., Wen X., Lingel A., de Sauvage F.J., Lazarus R.A., Scales S.J., Hymowitz S.G. (2010). Hedgehog pathway antagonist 5E1 binds hedgehog at the pseudo-active site. J. Biol. Chem..

[126] Teichman J., Dodbiba L., Thai H., Fleet A., Morey T., Liu L., McGregor M., Cheng D., Chen Z., Darling G. (2018). Hedgehog inhibition mediates radiation sensitivity in mouse xenograft models of human esophageal adenocarcinoma. PLoS ONE.

[127] Song Z., Yue W., Wei B., Wang N., Li T., Guan L., Shi S., Zeng Q., Pei X., Chen L. (2011). Sonic hedgehog pathway is essential for maintenance of cancer stem-like cells in human gastric cancer. PLoS ONE.

[128] O’Toole S.A., Machalek D.A., Shearer R.F., Millar E.K., Nair R., Schofield P., McLeod D., Cooper C.L., McNeil C.M., McFarland A. (2011). Hedgehog overexpression is associated with stromal interactions and predicts for poor outcome in breast cancer. Cancer Res..

[129] Coon V., Laukert T., Pedone C.A., Laterra J., Kim K.J., Fults D.W. (2010). Molecular therapy targeting Sonic hedgehog and hepatocyte growth factor signaling in a mouse model of medulloblastoma. Mol. Cancer Ther..

[130] Stanton B.Z., Peng L.F., Maloof N., Nakai K., Wang X., Duffner J.L., Taveras K.M., Hyman J.M., Lee S.W., Koehler A.N. (2009). A small molecule that binds Hedgehog and blocks its signaling in human cells. Nat. Chem. Biol..

[131] Carpenter R.L., Ray H. (2019). Safety and Tolerability of Sonic Hedgehog Pathway Inhibitors in Cancer. Drug Saf..

[132] Matevossian A., Resh M.D. (2015). Hedgehog Acyltransferase as a target in estrogen receptor positive, HER2 amplified, and tamoxifen resistant breast cancer cells. Mol. Cancer.

[133] Peacock C.D., Wang Q., Gesell G.S., Corcoran-Schwartz I.M., Jones E., Kim J., Devereux W.L., Rhodes J.T., Huff C.A., Beachy P.A. (2007). Hedgehog signaling maintains a tumor stem cell compartment in multiple myeloma. Proc. Natl. Acad. Sci. USA.

[134] Jeng K.S., Chang C.F., Lin S.S. (2020). Sonic Hedgehog Signaling in Organogenesis, Tumors, and Tumor Microenvironments. Int. J. Mol. Sci..

[135] Jeng K.S., Chang C.F., Sheen I.S., Jeng C.J., Wang C.H. (2023). Cellular and Molecular Biology of Cancer Stem Cells of Hepatocellular Carcinoma. Int. J. Mol. Sci..

[136] Wang F., Ma L., Zhang Z., Liu X., Gao H., Zhuang Y., Yang P., Kornmann M., Tian X., Yang Y. (2016). Hedgehog Signaling Regulates Epithelial-Mesenchymal Transition in Pancreatic Cancer Stem-Like Cells. J. Cancer.

[137] Choi H.S., Kim S.L., Kim J.H., Lee D.S. (2020). The FDA-Approved Anti-Asthma Medicine Ciclesonide Inhibits Lung Cancer Stem Cells through Hedgehog Signaling-Mediated SOX2 Regulation. Int. J. Mol. Sci..

[138] Zhang L., Li L., Jiao M., Wu D., Wu K., Li X., Zhu G., Yang L., Wang X., Hsieh J.T. (2012). Genistein inhibits the stemness properties of prostate cancer cells through targeting Hedgehog-Gli1 pathway. Cancer Lett..

[139] Fan P., Fan S., Wang H., Mao J., Shi Y., Ibrahim M.M., Ma W., Yu X., Hou Z., Wang B. (2013). Genistein decreases the breast cancer stem-like cell population through Hedgehog pathway. Stem Cell Res. Ther..

[140] Zhang Q., Cao W.S., Wang X.Q., Zhang M., Lu X.M., Chen J.Q., Chen Y., Ge M.M., Zhong C.Y., Han H.Y. (2019). Genistein inhibits nasopharyngeal cancer stem cells through sonic hedgehog signaling. Phytother. Res..

[141] Li E., Zhang T., Sun X., Li Y., Geng H., Yu D., Zhong C. (2019). Sonic hedgehog pathway mediates genistein inhibition of renal cancer stem cells. Oncol. Lett..

[142] Yu D., Shin H.S., Lee Y.S., Lee D., Kim S., Lee Y.C. (2014). Genistein attenuates cancer stem cell characteristics in gastric cancer through the downregulation of Gli1. Oncol. Rep..

[143] El-Rayes B.F., Philip P.A., Sarkar F.H., Shields A.F., Ferris A.M., Hess K., Kaseb A.O., Javle M.M., Varadhachary G.R., Wolff R.A. (2011). A phase II study of isoflavones, erlotinib, and gemcitabine in advanced pancreatic cancer. Investig. New Drugs.

[144] Takimoto C.H., Glover K., Huang X., Hayes S.A., Gallot L., Quinn M., Jovanovic B.D., Shapiro A., Hernandez L., Goetz A. (2003). Phase I pharmacokinetic and pharmacodynamic analysis of unconjugated soy isoflavones administered to individuals with cancer. Cancer Epidemiol. Biomark. Prev..

[145] Lauth M., Bergstrom A., Shimokawa T., Toftgard R. (2007). Inhibition of GLI-mediated transcription and tumor cell growth by small-molecule antagonists. Proc. Natl. Acad. Sci. USA.

[146] Srivastava R.K., Kaylani S.Z., Edrees N., Li C., Talwelkar S.S., Xu J., Palle K., Pressey J.G., Athar M. (2014). GLI inhibitor GANT-61 diminishes embryonal and alveolar rhabdomyosarcoma growth by inhibiting Shh/AKT-mTOR axis. Oncotarget.

[147] Tong W., Qiu L., Qi M., Liu J., Hu K., Lin W., Huang Y., Fu J. (2018). GANT-61 and GDC-0449 induce apoptosis of prostate cancer stem cells through a GLI-dependent mechanism. J. Cell. Biochem..

[148] Kurebayashi J., Koike Y., Ohta Y., Saitoh W., Yamashita T., Kanomata N., Moriya T. (2017). Anti-cancer stem cell activity of a hedgehog inhibitor GANT61 in estrogen receptor-positive breast cancer cells. Cancer Sci..

[149] Mansoori B., Mohammadi A., Shirjang S., Baradaran B. (2017). MicroRNAs in the Diagnosis and Treatment of Cancer. Immunol. Investig..

[150] Lee R.C., Feinbaum R.L., Ambros V. (1993). The C. elegans heterochronic gene lin-4 encodes small RNAs with antisense complementarity to lin-14. Cell.

[151] Mohammadi A., Mansoori B., Aghapour M., Shirjang S., Nami S., Baradaran B. (2016). The Urtica dioica extract enhances sensitivity of paclitaxel drug to MDA-MB-468 breast cancer cells. Biomed. Pharmacother..

[152] Asadzadeh Z., Mansoori B., Mohammadi A., Aghajani M., Haji-Asgarzadeh K., Safarzadeh E., Mokhtarzadeh A., Duijf P.H.G., Baradaran B. (2019). microRNAs in cancer stem cells: Biology, pathways, and therapeutic opportunities. J. Cell. Physiol..

[153] Bai H.Y., Liao Y.J., Cai M.Y., Ma N.F., Zhang Q., Chen J.W., Zhang J.X., Wang F.W., Wang C.Y., Chen W.H. (2018). Eukaryotic Initiation Factor 5A2 Contributes to the Maintenance of CD133(+) Hepatocellular Carcinoma Cells via the c-Myc/microRNA-29b Axis. Stem Cells.

[154] Mott J.L., Kurita S., Cazanave S.C., Bronk S.F., Werneburg N.W., Fernandez-Zapico M.E. (2010). Transcriptional suppression of mir-29b-1/mir-29a promoter by c-Myc, hedgehog, and NF-kappaB. J. Cell. Biochem..

[155] Tsukasa K., Ding Q., Miyazaki Y., Matsubara S., Natsugoe S., Takao S. (2016). miR-30 family promotes migratory and invasive abilities in CD133(+) pancreatic cancer stem-like cells. Hum. Cell.

[156] Krantz S.B., Shields M.A., Dangi-Garimella S., Bentrem D.J., Munshi H.G. (2010). Contribution of epithelial-mesenchymal transition to pancreatic cancer progression. Cancers.

[157] Munoz J.L., Rodriguez-Cruz V., Rameshwar P. (2015). High expression of miR-9 in CD133(+) glioblastoma cells in chemoresistance to temozolomide. J. Cancer Stem Cell Res..

[158] Owens A.E., de Paola I., Hansen W.A., Liu Y.W., Khare S.D., Fasan R. (2017). Design and Evolution of a Macrocyclic Peptide Inhibitor of the Sonic Hedgehog/Patched Interaction. J. Am. Chem. Soc..

[159] Yun T., Wang J., Yang J., Huang W., Lai L., Tan W., Liu Y. (2020). Discovery of Small Molecule Inhibitors Targeting the Sonic Hedgehog. Front. Chem..

[160] Tian S., Quan H., Xie C., Guo H., Lu F., Xu Y., Li J., Lou L. (2011). YN968D1 is a novel and selective inhibitor of vascular endothelial growth factor receptor-2 tyrosine kinase with potent activity in vitro and in vivo. Cancer Sci..

[161] Cao W., Li Y., Sun H., Yang C., Zhu J., Xie C., Li X., Wu J., Geng S., Wang L. (2021). Apatinib Suppresses Gastric Cancer Stem Cells Properties by Inhibiting the Sonic Hedgehog Pathway. Front. Cell Dev. Biol..

[162] Sneha S., Nagare R.P., Sidhanth C., Krishnapriya S., Garg M., Ramachandran B., Murhekar K., Sundersingh S., Ganesan T.S. (2020). The hedgehog pathway regulates cancer stem cells in serous adenocarcinoma of the ovary. Cell. Oncol..

[163] Koike Y., Ohta Y., Saitoh W., Yamashita T., Kanomata N., Moriya T., Kurebayashi J. (2017). Anti-cell growth and anti-cancer stem cell activities of the non-canonical hedgehog inhibitor GANT61 in triple-negative breast cancer cells. Breast Cancer.

[164] Miyazaki Y., Matsubara S., Ding Q., Tsukasa K., Yoshimitsu M., Kosai K., Takao S. (2016). Efficient elimination of pancreatic cancer stem cells by hedgehog/GLI inhibitor GANT61 in combination with mTOR inhibition. Mol. Cancer.

[165] Zhu Q., Shen Y., Chen X., He J., Liu J., Zu X. (2020). Self-Renewal Signalling Pathway Inhibitors: Perspectives on Therapeutic Approaches for Cancer Stem Cells. Onco Targets Ther..

[166] Omar A., Ruff P., Penny C. (2023). Inhibition of the Sonic Hedgehog Pathway using Small Molecule Inhibitors: Targeting Colon Cancer Stem Cells. Curr. Cancer Ther. Rev..

[167] Yao J., An Y., Wie J.S., Ji Z.L., Lu Z.P., Wu J.L., Jiang K.R., Chen P., Xu Z.K., Miao Y. (2011). Cyclopamine reverts acquired chemoresistance and down-regulates cancer stem cell markers in pancreatic cancer cell lines. Swiss. Med. Wkly..

[168] Meiss F., Andrlova H., Zeiser R. (2018). Vismodegib. Recent Results Cancer Res..

[169] Singh B.N., Fu J., Srivastava R.K., Shankar S. (2011). Hedgehog signaling antagonist GDC-0449 (Vismodegib) inhibits pancreatic cancer stem cell characteristics: Molecular mechanisms. PLoS ONE.

[170] Li W., Yang H., Li X., Han L., Xu N., Shi A. (2019). Signaling pathway inhibitors target breast cancer stem cells in triple-negative breast cancer. Oncol. Rep..

[171] Pietrobono S., Stecca B. (2018). Targeting the Oncoprotein Smoothened by Small Molecules: Focus on Novel Acylguanidine Derivatives as Potent Smoothened Inhibitors. Cells.

[172] Calcaterra A., Iovine V., Botta B., Quaglio D., D’Acquarica I., Ciogli A., Iazzetti A., Alfonsi R., Lospinoso Severini L., Infante P. (2018). Chemical, computational and functional insights into the chemical stability of the Hedgehog pathway inhibitor GANT61. J. Enzyme Inhib. Med. Chem..

[173] Ghirga F., Mori M., Infante P. (2018). Current trends in Hedgehog signaling pathway inhibition by small molecules. Bioorg. Med. Chem. Lett..

[174] Galperin I., Dempwolff L., Diederich W.E., Lauth M. (2019). Inhibiting Hedgehog: An Update on Pharmacological Compounds and Targeting Strategies. J. Med. Chem..

[175] Schaffner T.J., Skoner D.P. (2009). Ciclesonide: A safe and effective inhaled corticosteroid for the treatment of asthma. J. Asthma Allergy.

